# The bidirectional biological interplay between microbiome and viruses in periodontitis and type-2 diabetes mellitus

**DOI:** 10.3389/fimmu.2022.885029

**Published:** 2022-09-05

**Authors:** Boyu Tang, Caixia Yan, Xin Shen, Yan Li

**Affiliations:** ^1^ State Key Laboratory of Oral Diseases, National Clinical Research Center for Oral Diseases, West China Hospital of Stomatology, Sichuan University, Chengdu, China; ^2^ Department of Cariology and Endodontics, West China Hospital of Stomatology, Sichuan University, Chengdu, China

**Keywords:** microbiome in periodontitis, type-2 diabetes mellitus, the bidirectional immune regulation, bacterial metabolites, cytokines

## Abstract

Periodontitis was an inflammatory disease associated with a dysbiosis of the oral flora characterized by a chronic sustained inflammation inducing the resorption of alveolar bone and leading to tooth loss. Type 2 diabetes mellitus (T2D) was a metabolic disease caused by impaired insulin action. The oral microbiome played a crucial role in modulating both the innate and adaptive immune system during the trigger and exacerbation of periodontitis and T2D. The bidirectional relationship of T2D and periodontitis had been the focus of intensive research, but those were not well explored. In this commentary, an in-depth analysis of the changes of microbiome and bacterial metabolites in periodontitis with or without diabetes was described. The promotion of periodontitis to T2D might involve inflammatory factors/receptors, oxidative stress, microRNA and so on. The effect of diabetes on periodontitis might involve adipose factor pathway, AGE/RAGE and RANK/RANKL pathway etc. Generally, periodontitis and diabetes are closely related to the microecological-epithelial interaction, soft tissue degradation, bone coupling disorder, immune regulation and gene transcription. The viruses, including HBV, HCV, HSV-1, Coronavirus, HCMV, EBV, HIV, phageome and so on, played an important role in the development of T2D and periodontitis. An in-depth understanding of the relationship between microbiome and host was of great significance to clarify the bidirectional mechanisms, suggesting that the periodontitis or T2D remission will have a positive impact on the other.

## 1 Introduction

Periodontitis, a local inflammatory disease induced by oral microorganisms, could cause alveolar bone destruction and tooth loosening, which was the primary cause of tooth loss in adults. More data suggested that periodontitis was associated with systemic diseases such as diabetes, cardiovascular disease, respiratory disease, nervous system disease, tumor and so on (1). Local microbiota and host immune response were the most important links in the occurrence and development of periodontitis. Diabetes was mainly characterized by abnormal glucose and lipid metabolism, more than 90% of which were type 2 diabetes mellitus (T2D). It was estimated that the number of diabetes patients in the world will reach 642 million by 2040 (2). Periodontitis was considered to be the sixth complication of diabetes, and the occurrence of periodontitis could increase the risk of diabetes by 27-53% (3). The relationship between periodontitis and diabetes and the mechanism of flora on immune regulation had always been the focus of the stomatology research. In clinic, it was generally believed that periodontal microbiome played an important role in the regulation of immune system and substance metabolism in the process of triggering and worsening of diabetes.

## 2 Oral microbiome

The occurrence and development of periodontitis were closely related to host immune response, local microbiota and their metabolites. The local immune response and periodontal flora were in dynamic balance under physiological conditions. When the colonization of key pathogens or changes in microbiota happened, it could lead to an increase in the pathogenicity of the whole community and the destruction of local tissue homeostasis (3, 4). At this time, the host immune response was overactivated, leading to the release of inflammatory factors and immune cell infiltration, playing a “bridge” role between diabetes and periodontitis.

### 2.1 Oral microbiome in periodontitis

**Figure 1:**
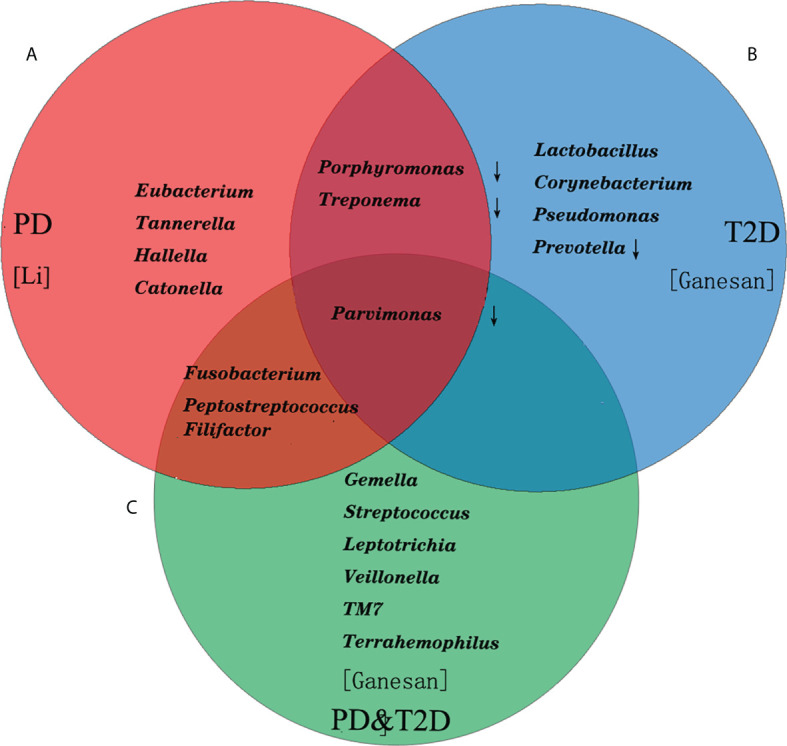
The composition of the top 10 bacterial genera in relative abundance in patients with periodontitis and T2D. **(A)** The *Tannerella*, *Catonella*, *Eubacterium*, *Parvimonas*, *Hallella* and so on were closely related to periodontitis. **(B)** The *Lactobacillus*, *Corynebacterium*, *Pseudomonas* in T2D increased significantly. **(C)** The *Fusobacterium*, *Peptostreptococcus*, *Filifactor* shared with PD, and *Parvimonas* shared with PD and T2D.

Periodontitis was one of the common oral diseases. It was believed that the special inflammation caused by “red complex” (*Porphyromonas gingivalis*, *Tannerella forsythensis* and *Treponema denticola*) which was the main cause in the past. Microecological theory held that periodontitis was a non-specific inflammation caused by flora imbalance and host immune response in recent years. The difference of microbial composition of subgingival plaque between patients with periodontitis and healthy people was analyzed by 16S rRNA sequencing. It was found that in patients with periodontitis, *Proteobacteria, Firmicutes* and *Actinomycetes* decreased. However, *Bacteroides* and *Fusobacteria* increased, especially the *Synergistetes* and *Spirochaetes* increased significantly (4, 5). At the genus level, patients with periodontitis had relatively high abundance of *Porphyromonas*, *Fusobacterium*, *Treponema*, *Nematogen*, and *Peptostreptococcus* (6, 7). The recent study also found that 39 species of bacteria, such as *Tannerella*, *Catonella*, *Eubacterium*, *Parvimonas*, *Hallella* and so on, were closely related to periodontitis (**Figure 1A**) (8). At the species level, red complex and *Fusobacterium nucleatum* (*F. nucleatum*) were the main bacteria causing periodontitis. *Filifactor alocis* was also often detected in subgingival plaque of patients with periodontal disease, which could participate in periodontal tissue destruction through antioxidative stress and regulation of amino acid metabolism (9). Functional microarray HumiChip data showed that genes involved in amino acid metabolism, glycosaminoglycan metabolism, pyrimidine metabolism and virulence factors were abundant in periodontitis. However, there was no significant difference in carbohydrate metabolism (8) (**Table 1**).

**Table 1:** The functional potentials in PD and PD with T2D communities.

Functional potentials	PD vs PD with T2D	Reference
virulence genes	colonization factor, fimbriae*, hly, iuc, pap, pilin* and *srt*	PD [Li]
Amino acid metabolism	Lysinedecarboxylase; L-alanine dehydrogenase; biosynthetic arginine decarboxylase, PLP-binding; argininosuccinate lyase	
glycosaminoglycan degradation	N-acetyl-D-galactosamine-4-sulfate 4-sulfohydrolase, b-N-acetyl-D-hexosaminide N-acetylhexosaminohydrolase, N-acetyl-D-glucosamine-6-sulfate 6-sulfohydrolase;	
Pyrimidine metabolism	thymidine phosphorylase, uridine phosphorylase;	
Organic acids	b-ketoacyl-ACP synthase III, butyrate kinase, butyrate-acetoacetate CoA-transferase;	
Carbohydrate degradation	Not significant	
Lipid metabolism	either lipid metabolism, arachidonic acid metabolism	[Shi]
carbohydrate metabolism	inositol phosphate metabolism	
Signal pathway*	[PD with T2D] Two component system	
cell motility*	[PD, PD with T2D both] bacterial motility, flagellar assembly, bacterial chemotaxis	

*Prevalent in the periodontitis statein T2D and non-diabetic (ND).

PLP, pyridoxal-5’-phosphate; ACP, β-ketoacyl-acyl-carrier-protein.

### 2.2 Oral microbiome in periodontitis and diabetes

It was believed that hyperglycemia environment could selectively promote the rapid growth of some pathogenic bacteria in subgingival flora, thus increasing the susceptibility to periodontitis and periodontal tissue destruction in patients in the past (10, 11). Ganesan et al. found that *Lactobacillus*, *Corynebacterium*, *Pseudomonas* in T2D (**Figure 1B**) increased significantly. However, the relative abundance of four species of bacteria (*Porphyromonas, Treponema*, *Prevotella* and *Parvimonas*) decreased (12). Demmer et al. found that there was a negative correlation between insulin resistance of T2D and *Actinomyces*, *Proteus* and *Firmicutes* in subgingival plaque microbiome (13). Recent studies had found that there was no difference in the effect of T2D on the microflora of subgingival plaque in patients with periodontitis, only the change of bacteria, such as the abundance of “red complex” bacteria, *F. nucleatum* and *Capnocytophaga sputigena* (*C. sputigena*) (14). *C. sputigena* was a kind of glycolytic bacteria, which could digest glucose in gingival crevicular fluid. As a consequence, the content was higher in patients with T2D and periodontitis. Other studies had shown that the main difference of periodontal microorganisms between T2D and periodontitis lied in the pathogenicity of bacteria at the level of bacteria, or the different response of the body to the flora (14). In addition, periodontitis has also been associated with cardiovascular diseases and considered a cardiovascular risk factor (15). The periodontitis-associated pathogens could enhance the development of T2D by exacerbating the dyslipidemia, the most important risk factor for atherosclerosis (16, 17).

At the same time, the subgingival dominant flora of periodontitis patients with T2D (PD&T2D) had been well investigated, and their results showed that the *Gemella*, *Streptococcus*, *Leptotrichia*, *Veillonella*, *TM7* and *Terrahemophilus* were specific, *Fusobacterium*, *Peptostreptococcus*, *Filifactor* shared with PD, and *Parvimonas* shared with PD and T2D (**Figure 1C**). Some studies had also reported a significant increase in the absolute abundance of *C. sputigena* (5), and other studies had reported different dominant bacteria (13, 18). There was still no consensus on the specific changes of oral bacteria caused by diabetes though microbial sequencing technology was widely used. Considering the degree and duration of hyperglycemia, diet and oral hygiene and drugs and other confounding factors affected the results, as well as the limitations of sequencing methods, could also lead to deviation (19). Some researchers had proposed three possible pathways for diabetes to affect periodontal flora: 1. the increase of glucose levels in the saliva of diabetic patients would promote the growth of some bacterial species; 2. diabetes would lead to oral dehydration and reduce microbial diversity; 3. hyperglycemia might lead to oral acidification and disrupt oral microbiota (2).

## 3 Bacterial metabolites

The virulence factors of the microbiome were enriched, and the abnormal metabolism of sugar, lipid and protein could promote the imbalance of immunity in the periodontal region in the state of periodontitis. However, the accumulation of glycosylated metabolites, fat factors and the changes of metabolic pathways regulated the host’s response to periodontal pathogens in the state of diabetes.

### 3.1 Microbiome metabolism in periodontitis

Periodontal pathogens could produce a large number of virulence factors, such as fimbriae, hemolysin, colonization factor and so on, through functional gene detection and clinical research verification (**Table 1**). Some proteases synthesized and secreted by periodontal microorganisms could be involved in protein degradation into amino acids and peptides, because their concentration in gingival crevicular fluid (GCF) of patients with periodontitis was significantly higher than that of healthy people (20). These metabolites could be used as energy repositories for some special strain’s dependent on oligopeptides (19). Proteoglycan was a linear macromolecule formed by covalent and non-covalent bonds between proteins and polysaccharides in the matrix, which could form molecular sieves with tiny pores and limit the diffusion of harmful substances. A significant increase in its concentration was detected in patients with periodontitis (21). In addition, organic acid metabolites such as butyric acid produced by bacteria were enriched in periodontium. Butyric acid could be produced by bacteria metabolizing amino acids, hexose or pentose *via* anaerobic glycolysis. The concentration of butyric acid in GCF of patients with periodontitis was significantly higher than that of healthy controls, which was positively correlated with the severity of periodontal disease (22). High concentration of butyric acid might destroy the structure and function of periodontal epithelial tissue and play an important role in the occurrence and development of periodontitis. Moreover, the genes related to butyric acid synthesis, such as butyrate kinase and butyrate acetyl-CoA transferase coding genes, were enriched in the subgingival plaque of patients with periodontitis (8).

### 3.2 Microbiome metabolism of periodontitis with diabetes mellitus

Recently, the research found that there were metabolic pathways related to cell movement (bacterial movement, flagellar assembly and bacterial chemotaxis) and signal transduction pathways (two-component regulatory system) involved in the pathogenesis of periodontitis microorganisms, regardless of whether they were associated with diabetes or not (14) (**Table 1**). There were three pathways that didn’t exist in T2D but only existed in periodontitis, including lipid metabolism (butyric acid metabolism) and two phosphate metabolic pathways of inositol (conversion between pentose and glucuronic acid, ascorbic acid and aldehydes metabolism). Microbial butyric acid had been used as the marker of periodontitis, and butyric acid could affect the body’s sensitivity to insulin (23). The study found that the oral environment in periodontitis with poor blood glucose control was conducive to the survival of sugar-fermenting bacteria, especially those related to the production of propionic acid and succinic acid. However, the number of bacteria related to the formation of butyric acid and pyruvate decreased (24, 25). Ascorbic acid and fat metabolic pathways had also been shown to be associated with periodontitis and T2D.

The combination of blood glucose with proteins, lipids and nucleic acids could increase the level of advanced glycation end products (AGEs). The levels of serum total cholesterol, triglyceride, low density lipoprotein and free fatty acids in diabetic patients also increased along with the severity of periodontitis (24, 26, 27). The hyperglycemia of T2D patients could also increase the AGEs of periodontal tissue, then combine with RAGEs on the surface of immune cells, and induce the release of inflammatory cytokines, thus promoting periodontal destruction (28, 29). It could also promote the increase of gram-negative anaerobes, which could be used as evidence to explain the increased risk of periodontitis in patients with diabetes (30).

In addition, obesity-related metabolic syndromes such as insulin resistance, insulin sensitivity and dyslipidemia were important factors in the pathogenesis of periodontal disease. Adipocytes could release pro-inflammatory cytokines: IL-6, IL-1 and tumor necrosis factor α (TNFα); adipokines; other intracellular signals: reactive oxygen species, which directly affected the pathogenesis of periodontal tissue inflammation and progress (31). Adipokines, the adipocyte-derived bioactive substances, were molecules produced and secreted by adipocytes, which were mainly composed of leptin, resistin, lactone and adiponectin. Leptin could inhibit the production of insulin through negative feedback. Resistin could induce insulin resistance and cause disorders of glucose and lipid metabolism (32). However, adiponectin has the protective effects of anti-inflammation and anti-diabetes (24).

## 4 Immune regulation of periodontitis microbiome and diabetes mellitus

The two-way relationship between diabetes and periodontitis was mainly manifested in that untreated periodontitis could stimulate the production of antibodies by bacteria, induce the production of proinflammatory cytokines and then act on immune cells, thus promoting insulin resistance. In addition, uncontrolled diabetes could aggravate the destruction of bone and supporting tissue, exacerbate the progress of periodontitis by changing periodontal microbiota, causing local immune dysfunction, etc. However, there was not enough evidence to explain the mechanism by which periodontitis microbiome affected the disease state of diabetes. It was inferred that it might be the secretion of many inflammatory factors and immune cell infiltration caused by periodontal microbial infection, which acted as a bridge between diabetes and periodontitis (27). In addition, it might also be systemic inflammation caused by direct migration and colonization of periodontal microorganisms to distant organs (33).

### 4.1 Immunomodulatory effect of periodontitis microbiome on diabetes

**Figure 2:**
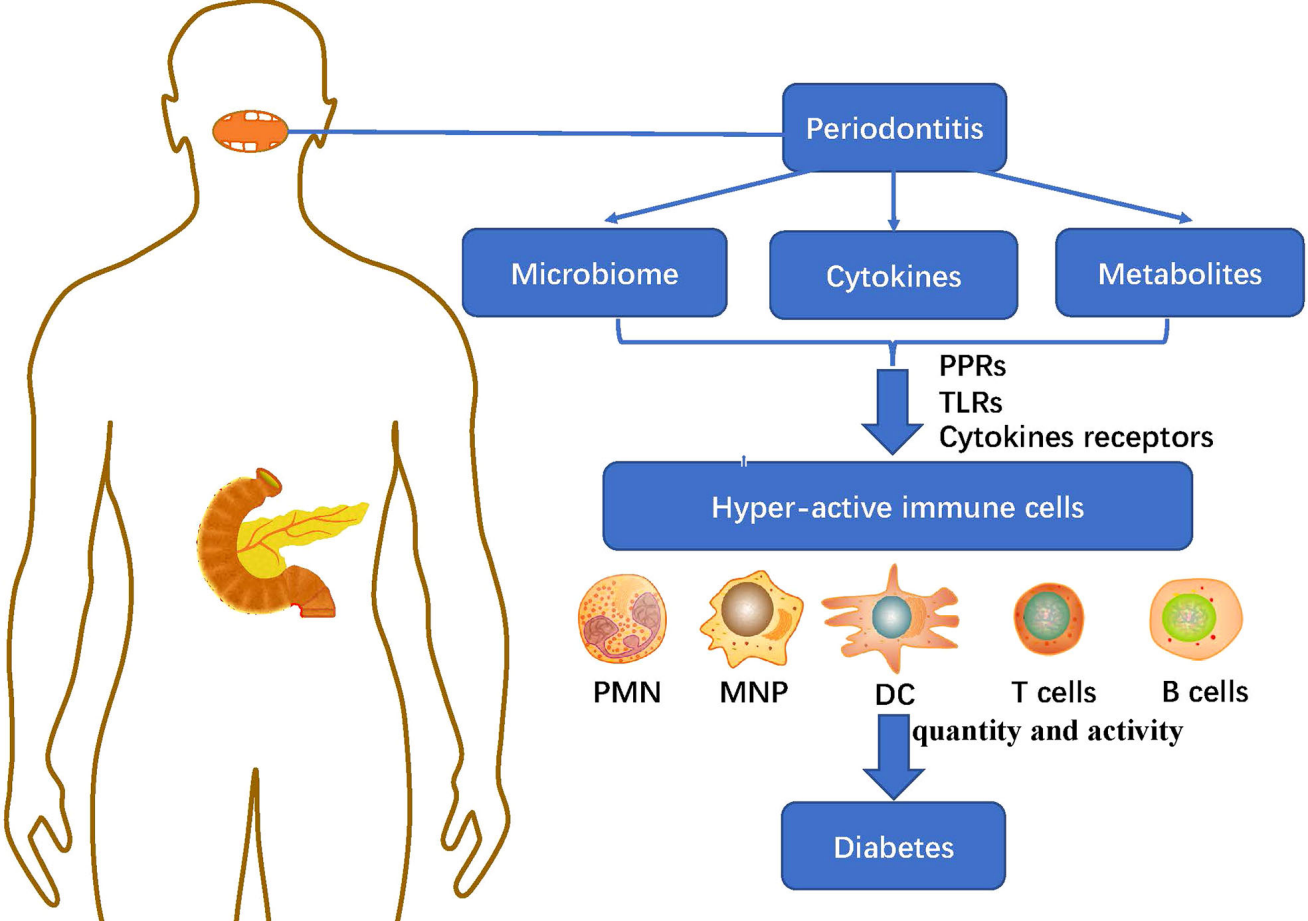
The immunoregulatory effect of periodontitis microbiome and metabolites on diabetes: The imbalance of periodontal microbiome and the accumulation of cytokines and metabolites in periodontitis mediate the recruitment, activation and differentiation of specific immune cells including: PMN, MNP, DC, T cells and B cells, through PRRs, TLRs and cytokines receptors, causing long-term low-grade inflammatory state of the host and promoting the progress of diabetes. PMN, polymorphonuclear neutrophil; MNP, mononuclear phagocytic cells; DC, dendritic cells; PRRs, pattern recognition receptors; TLRs, Toll-like receptors.

The immune system was involved in maintaining the dynamic balance between the host and the symbiotic microflora. However, the imbalance of periodontal flora and the accumulation of metabolites would excessively activate the host immune response (**Figure 2**), resulting in the secretion and cascade amplification of a large number of pro-inflammatory cytokines. Besides, it could mediate the recruitment, activation and differentiation of specific immune cells and eventually lead to periodontal and distant organ inflammation and tissue destruction, causing long-term low-grade inflammatory state of the host and promoting the progress of a variety of diseases such as diabetes (32).

#### 4.1.1 Cytokines

The network of cytokines in the pathogenesis of periodontitis was very complex. In addition, there was great heterogeneity in the inflammatory response among individuals. Periodontitis could promote the secretion of a variety of inflammatory factors, including TNF-α, IL-1β, IL-6, IL-8, monocyte chemoattractant protein-1, chemokine-5, intercellular adhesion molecule-1 and vascular cell adhesion molecule-1 (34). Some of the above inflammatory factors had clear pro-inflammatory and tissue destruction effects, such as IL-1, IL-6 and TNF-α. The binding of TNF-α to its receptor would induce pancreatic β-cells apoptosis in a time-dependent and dose-dependent manner (35). In addition, the other part was closely related to the differentiation and maturation of specific immune cell subsets, which could cooperatively activate and recruit specific immune cells and initiate adaptive immune response. In addition, the high expression of C-reactive protein (CRP) could be detected in the serum of patients with periodontitis. CRP could destroy the intracellular insulin signal and lead to the accumulation of insulin resistance, hyperglycemia and advanced glycation end products. The increase of CRP level could also be detected in the serum of T2D mice with periodontitis, which not only promoted periodontal inflammation, but also accelerated the development of diabetes (36, 37).

#### 4.1.2 Innate immunity

In fact, innate immune response was the first line of defense of gingival epithelium against pathogenic microorganisms. It could identify invading microorganisms and trigger an immune response to eliminate them. Innate immune cells included the neutrophil polymorphonuclear leukocytes (PMNs), monocytes, macrophages, dendritic cells (DCs), natural killer cells and so on. They mainly recognized pathogen-related molecular patterns of pathogenic microorganisms through a series of pattern recognition receptors (PRRs) and promoted intracellular signal transduction. As one of the main effector cells of periodontal innate immune, PMNs could kill and scavenge periodontal pathogenic microorganisms (24). The number of PMNs in jaw bone marrow of periodontitis mouse model increased significantly. The detection of monocytes found that poor blood glucose control in T2D patients led to a more than two-fold increase in the proportion of monocytes, and the level of hemoglobin A1c (HbA1c) was positively correlated with the proportion of monocytes (38). Macrophages could differentiate from infiltrating monocytes in periodontitis. Macrophages could be polarized into M1 and participated in the elimination of microorganisms in the initial stage of periodontitis. In the recovery stage of periodontitis, macrophages could be transformed from M1 to M2 and apoptotic PMN cells could be eliminated through efferocytosis (26). Induced by *P. gingivalis*, macrophages played a role in insulin resistance in T2D patients by triggering inflammatory responses such as TNF-α and IL-6. DCs cells had the potential to prevent the onset of diabetes and assist in maintaining the balance of immune cells. Periodontitis significantly reduced the number of CD11b+DC cells in cervical lymph nodes and spleen in T2D mice (28).

#### 4.1.3 Adaptive immunity

After being stimulated by microorganisms, innate immune cells and periodontal tissues could secrete a series of cytokines, which could induce immature T cells of CD4+ to differentiate into different subsets, including helper T cells: Th1, Th2 and Th17 (27, 39). Different T cell subsets and related inflammatory factors played different roles in periodontal immunity, such as enhancing the control of periodontal flora and mucosal barrier, and promoting the destruction of soft tissue and hard tissue. In addition, the balance and differentiation of T cell subsets could also affect the progress and prognosis of periodontitis (27). The role of T cells was still controversial, and there was little specific evidence to reveal the function of specific CD4+T cell subsets in periodontitis. B cells were also involved in the inflammatory process of periodontal tissue. The presence of periodontitis could increase the number of T and B cells in gingival tissue and blood of T2D patients, B cells could further induce osteoclast differentiation, and the proinflammatory cytokine profile secreted by B cells of periodontitis patients was similar to that of T2D patients, such as TNF-α and IL-1β, which could promote periodontitis and diabetes (20, 40). Recent studies had shown that the colonization of *P. gingivalis*, *F. nucleatum* and *P. intermedius* could increase the activity of adaptive immune CD4+T, CD8+T and B cells in cervical lymph nodes and blood of T2D mice (28). Lipopolysaccharide (LPS) produced by *P. gingivalis* could reduce the proportion of B cell apoptosis by activating TLR2-and TLR4-dependent pathway. Some researchers speculated that memory B cells could also secrete inflammatory factors to regulate periodontal soft tissue and bone tissue-related inflammation. However, it still needed to be further confirmed (29).

### 4.2 Immune regulation of diabetes mellitus on periodontitis microbiome

**Figure 3:**
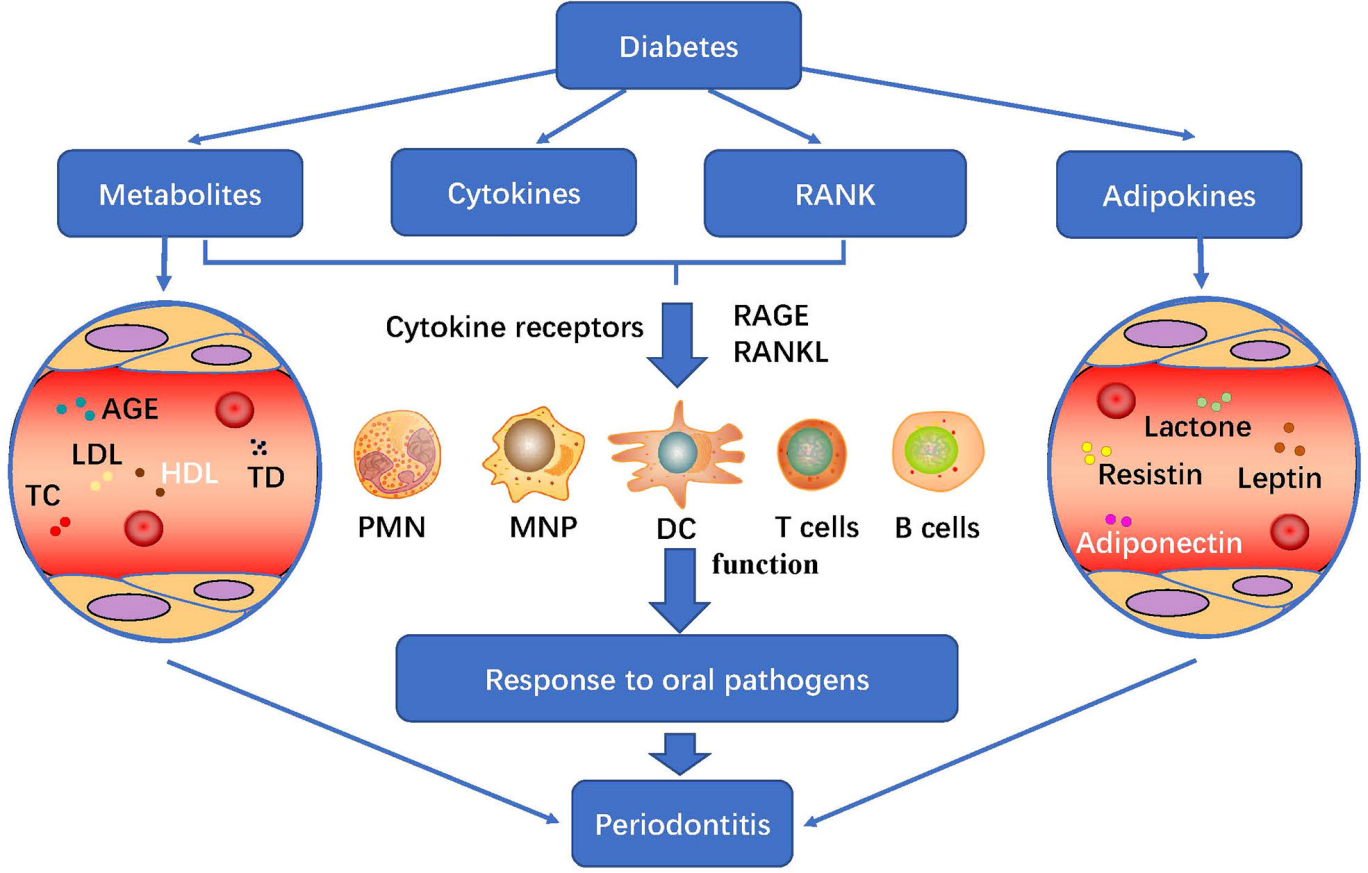
Diabetes metabolism and its immune regulation effect on periodontitis: diabetes regulates the host’s response to periodontal pathogens by changing its structure and metabolites AGE, fat factor, cytokines, bone metabolism and immune response, and participate in the immune regulation of diabetes to periodontitis microflora. PMN, polymorphonuclear neutrophil; MNP, mononuclear phagocytic cells; DC, dendritic cells; PRRs, pattern recognition receptors; TLRs, Toll-like receptors; RANK, Receptor activator of NF-κB; AGEs, advanced glycation end products; LDL, low density lipoprotein; HDL, high density lipoprotein; TC, total cholesterol.

Diabetes could regulate the host’s response to periodontal pathogens by changing its structure and metabolites AGE, fat factor (see part 2.2), cytokines, bone metabolism and immune response, and participate in the immune regulation of diabetes to periodontitis microflora (**Figure 3**). In addition, T2D increased periodontal inflammation and alveolar bone loss in the host. Receptor activator of NF-κB (RANK) binded to its ligand and participated in bone metabolism, which coincided with the increase in the number and activity of osteoclasts (32, 41).

#### 4.2.1 Cytokines

Interleukin was a key medium in the link between periodontitis and diabetes. It had been found that there were high levels of IL-1β, IL-6 and IL-17 in the serum of patients with periodontitis or T2D. These cytokines were related to the destruction of intracellular insulin signal and production of insulin resistance. The high expression of these cytokines could also be detected when periodontitis coexisted with T2D (42, 43). A clinical study of 130 subjects found that there was no difference in the levels of serum IL-17, IL-1β and TNF-α between patients with T2D and periodontitis or periodontitis only (44, 45). However, the ratio of pro-inflammatory to anti-inflammatory factors, such as TNF-α/IL-4 and IL-6/IL-4, was significantly increased. As a consequence, the researchers suggested that serum cytokine ratio rather than absolute cytokine levels could be used as an indicator of systemic pre-inflammatory state in patients with T2D and periodontitis. In addition, the expression of IL-34 in GCF and serum of patients with T2D and periodontitis was also significantly increased. IL-34 could increase the levels of IL-6 and chemokines in human whole blood (23). Together, they could aggravate the inflammatory state of the whole body. In addition, IL-34 also played an important role in the process of bone fracture induced by RANKL, and was also related to insulin resistance (46). Other cytokines also changed in the presence of both diseases, such as increased expression of IFN-γ in GCF of patients with periodontitis and diabetes.

#### 4.2.2 Innate immunity

Exploring how diabetes affected the immune response of periodontal tissue to specific periodontal pathogens contributed to clarify the mechanism of diabetes promoting periodontitis. The combination of RAGE receptors expressed by macrophages and AGEs produced by T2D could activate monocytes, macrophages and endothelial cells, and induce the release of proinflammatory cytokines (47). Higher levels of CD14+CD16+ monocytes could be detected in peripheral blood of patients with poorly controlled T2D periodontitis (38). However, there was no direct evidence for the role of monocytes and macrophages in patients with T2D periodontitis. In addition, the number of neutrophils in GCF increased significantly in patients with T2D periodontitis and induced high periodontal inflammation. Moreover, studies had shown that the increase of high inflammatory phenotype of monocytes and the changes of adhesion, chemotaxis and phagocytosis of neutrophils would lead to delayed clearance of bacteria, continuous accumulation of periodontal anaerobic microorganisms and oral flora imbalance (48).

#### 4.2.3 Adaptive immunity

Adaptive immunity was also important for the progression of diabetes and periodontitis. However, the evidence for changes in lymphocyte function in patients with diabetic periodontitis was also limited. Immunohistochemistry showed that Th17 and regulatory T cells (Tregs) increased in patients with periodontitis and diabetes (27, 49). They could be recognized by monocytes under the mediation of TLR2/4, and then activated CD4+T cells and Th17/IL-17 axis in gingival tissue, and induced the increase of IL-17 secretion when periodontal pathogens invaded the diabetic body (43). Treg cells were the main regulators of immune tolerance, which could inhibit the induction, proliferation and effector function of Th17 cells, restore immune balance, and play a core role in controlling the disease progression of T2D (42, 44, 45). In recent studies, single cell sequencing analysis of gingiva from healthy people, patients with chronic periodontitis and patients with T2D periodontitis showed that the gingival immune cells were mainly T cells, B cells were relatively rare, and there was no significant difference in the number of subtypes among different populations. According to the relative proportion of cells from high to low were CD4+T cells (about 25%), CD8+T cells (about 20%) and B cells (about 8%). However, the function of immune cells was different. For example, myeloid cells in gingival tissue of patients with T2D periodontitis could produce higher levels of IFN-γ and IL-10, while CD4+T could produce higher levels of IL-8. It was suggested that the difference in the function of gingival immune cells might be the root cause of the increased risk and severity of patients with T2D periodontitis (50).

## 5 Viruses in periodontitis and type-2 diabetes mellitus

### 5.1 Viruses in type-2 diabetes mellitus

T2D, a complex metabolic disease, had been shown to involve alteration of the oral and gut microbiota. Previous studies had primarily focused on changes in the bacterial microbiome, while ignoring the virus infection and alteration of virome in type-2 diabetes mellitus. Actually, the link between viruses and type 2 diabetes was stronger than we thought. A study had investigated changes in the extracellular phageome from fecal samples with or without T2D. The abundance of 58 species of phage (including *Brochothrix*_phage_NF5, *Enterococcus*_phage_phiFL2A, *Streptococcus*_phage_PH10, and *Streptococcus*_phage_7201) was significantly different between T2D patients and nondiabetic controls, especially, the phages specific to *Enterobacteriaceae* hosts (51). Another larger study also had showed that a decreased gut viral richness and diversity was found in obese subjects with T2D (ObT2) compared with lean controls. Besides, 17 differentially abundant viruses were identified between the two groups (52). In addition, the chronic hepatitis C virus (HCV) infection was associated with an increased risk of developing insulin resistance and T2D had been shown in some cross-sectional and longitudinal studies, *via* affecting glucose metabolism and interfering with host innate immune response (53). On the contrary, insulin resistance (IR) and T2D accelerated the histological and clinical progression of chronic hepatitis C and reduced the early and sustained virological response to interferon-alpha-based therapy. The association of virus infection in T2D was also common. A community-based study (9621 participants) had shown that HBV/HCV coinfection was significantly correlated with blood glucose levels and 28% of participants with HBV/HCV coinfection developed T2D (54). Furthermore, the association of virus infection (HCV and HSV-1) in T2D and Alzheimer’s disease (AD) had been reported in few recent studies (55).

At present, the outbreak of COVID-19 (Coronavirus disease 2019) infection had been defined as a global pandemic by the World Health Organization (WHO), and the COVID-19 patients with T2D had higher rates of severe illness and mortality than those without T2D (56). After COVID-19 invaded the human body, it bound to the angiotensin-converting enzyme 2 (ACE2) receptors of β cells, adipocytes and liver cells, directly damaging islet beta cells, fat, and liver cells, further aggravating insulin resistance (57). COVID-19 could bind to the ACE2 receptors of islet alpha cells to reduce the protective effect of ACE2/Ang (1–7)/Mas in β cells, aggravating the damage and dedifferentiation of β cells (58). *In vivo*, the inflammatory storm and systemic inflammatory response syndrome caused by COVID-19 could activate Toll-like receptors, imbalance of Th1/Th2 and Th17/Tregs, leading to pancreatic β-cell damage and the occurrence of acute diabetes (59, 60). On the contrary, the complement system mediated B lymphocytes producing of specific antibodies against the virus. For the balance impairments of humoral immune function in diabetic patients, the levels of complement C3 and C4 decreased. Then, the insufficient production of immunoglobulins might increase the risk of COVID-19 infection.

### 5.2 Viruses in periodontitis

A large number of viral genome replications could be detected in the periodontal sites of patients with periodontal disease, including herpes virus, human immunodeficiency virus (HIV), hepatitis virus (HBV and HCV), influenza virus etc. Epstein-barr virus (EBV) and human cytomegalovirus (HCMV) had higher detection rates in chronic and invasive periodontitis, indicating that herpes virus played a role in severe periodontitis (61). In addition to direct damage to cells and interfere with host immune system, virus infection in periodontitis could also act in synergy with periodontal pathogens, such as *P. gingivalis*. The detection and comparison of *P. gingivalis* in HIV-1 positive patients and healthy controls had showed that the activity of *P. gingivalis* was more active in the former. Butyric acid, a metabolite of *P. gingivalis*, could act as a negative regulator of the enzymatic activity of histone deacetylase (HDAC). Butyric acid inhibited catalytic action of HDAC and induced transcription of silenced genes including HIV-1 provirus, greatly reactivating the latently-infected HIV-1 (62). Butyric acid also activated latent EBV *via* promoting ZEBRA expression of EBV provirus (63). While promoting related viral replication, periodontal pathogens could promote their own proliferation with the help of virus, leading to the prolongation of periodontitis.

### 5.3 Viruses in periodontitis and type-2 diabetes mellitus

The virus infection in T2D and periodontitis had been reported in few recent studies. Poor glycemic control in type 2 diabetic subjects had a higher frequency of EBV in shallow periodontal pockets (64). Another cross-sectional study involved a total of 120 patients had shown that EBV-1 was predominant with poor glycemic status patients and the most significant levels of EBV-1 were detected in periodontitis with T2D (65). In addition, the increase of microRNA-146a and microRNA-155 in oral cavity induced by T2D and periodontitis was predicted to upregulate the expression of ACE2, the essential SARS-CoV-2 entry receptors, modulating host antiviral response. And it might suggest that T2D with periodontitis patients had a higher chance of infection (66).

In conclusion, viral infection played an important role in the development of T2D and periodontitis, especially in the former. The virus infection was more likely to occur when there were two diseases at the same time. In the process of diagnosis and treatment of related diseases, the potential role of viruses should be fully considered, and the combined treatment of bacterial and viral infections would be another new idea for the treatment of periodontitis and T2D when necessary.

### 6 Prospect

The relationship between diabetes and periodontitis highlighted the importance of early diagnosis in the later treatment. The control of blood sugar could improve the periodontal condition of diabetic patients, and the treatment of periodontal infection could reduce the incidence of diabetic complications. Although we had understood the interaction of these two diseases in the past few years, most of this knowledge was descriptive. At present, the research on the relationship between periodontitis and diabetes was mainly focused on the screening of differential microbiota, the changes of immune regulation and substance metabolism. The promotion of periodontitis to diabetes might involve inflammatory factors/receptors, oxidative stress, microRNA and other mechanisms to be clarified; the effect of diabetes on periodontitis might involve adipose factor pathway, AGE/RAGE and RANK/RANKL pathway and so on. Generally speaking, the effects of periodontitis and diabetes on microecological-epithelial interaction, soft tissue degradation, bone coupling disorder, immune regulation and gene transcription needed to be further studied. Furthermore, in the process of diagnosis and treatment of periodontitis and T2D, the potential role of viruses should be fully considered. It intended to accurately control specific immune cells or inflammatory factors to achieve the purpose of disease cure in the future.

## Author contributions

BT wrote the manuscript and contributed to literature review. CY contributed to the manuscript writing. XS contributed to figure production. YL designed the literature review. All authors contributed to the article and approved the submitted version.

## Funding

This study was supported by the National Natural Science Foundation of China (grant No.: 81771085), Key Projects of Sichuan Provincial Department of Science and Technology (2020YFSY0008), Natural Science Foundation of Sichuan Province (2022NSFSC1507), Research and Develop Program, West China Hospital of Stomatology Sichuan University (RD-02-202112).

## Conflict of interest

The authors declare that the research was conducted in the absence of any commercial or financial relationships that could be construed as a potential conflict of interest.

## Publisher’s note

All claims expressed in this article are solely those of the authors and do not necessarily represent those of their affiliated organizations, or those of the publisher, the editors and the reviewers. Any product that may be evaluated in this article, or claim that may be made by its manufacturer, is not guaranteed or endorsed by the publisher.

## References

[1] Liccardo D Cannavo A Spagnuolo G Ferrara N Cittadini A Rengo C. Periodontal disease: A risk factor for diabetes and cardiovascular disease. Int J Mol Sci (2019) 20(6):1414. doi: 10.3390/ijms20061414 PMC6470716 30897827

[2] Saeb ATM Al-Rubeaan KA Aldosary K Udaya Raja GK Mani B Abouelhoda M. Relative reduction of biological and phylogenetic diversity of the oral microbiota of diabetes and pre-diabetes patients. Microb Pathog (2019) 128:215–29. doi: 10.1016/j.micpath.2019.01.009 30625362

[3] Norhammar A Kjellström B Habib N Gustafsson A Klinge B Nygren Å. Undetected dysglycemia is an important risk factor for two common diseases, myocardial infarction and periodontitis: A report from the PAROKRANK study. Diabetes Care (2019) 42(8):1504–11. doi: 10.2337/dc19-0018 31182493

[4] Zhang Y Wang X Li H Ni C Du Z Yan F. Human oral microbiota and its modulation for oral health. Biomedicine Pharmacotherapy (2018) 99:883–93. doi: 10.1016/j.biopha.2018.01.146 29710488

[5] Park OJ Yi H Jeon JH Kang SS Koo KT Kum KY. Pyrosequencing analysis of subgingival microbiota in distinct periodontal conditions. J Dental Res (2015) 94(7):921–7. doi: 10.1177/0022034515583531 25904141

[6] Farina R Severi M Carrieri A Miotto E Sabbioni S Trombelli L. Whole metagenomic shotgun sequencing of the subgingival microbiome of diabetics and non-diabetics with different periodontal conditions. Arch Oral Biol (2019) 104:13–23. doi: 10.1016/j.archoralbio.2019.05.025 31153098

[7] Kirst ME Li EC Alfant B Chi Y-Y Walker C Magnusson I. Dysbiosis and alterations in predicted functions of the subgingival microbiome in chronic periodontitis. Appl Environ Microbiol (2015) 81(2):783–93. doi: 10.1128/AEM.02712-14 PMC4277562 25398868

[8] Li Y He J He Z Zhou Y Yuan M Xu X. Phylogenetic and functional gene structure shifts of the oral microbiomes in periodontitis patients. ISME J (2014) 8(9):1879–91. doi: 10.1038/ismej.2014.28 PMC4139721 24671083

[9] Aruni AW Mishra A Dou Y Chioma O Hamilton BN Fletcher HM. Filifactor alocis–a new emerging periodontal pathogen. Microbes Infect (2015) 17(7):517–30. doi: 10.1016/j.micinf.2015.03.011 PMC4485945 25841800

[10] Sabharwal A Ganley K Miecznikowski JC Haase EM Barnes V Scannapieco FA. The salivary microbiome of diabetic and non-diabetic adults with periodontal disease. J Periodontology (2019) 90(1):26–34. doi: 10.1002/JPER.18-0167 29999529

[11] Silva DNA Casarin M Monajemzadeh S Bezerra BB Lux R Pirih FQ. The microbiome in periodontitis and diabetes. Front Oral Health (2022) 3:859209. doi: 10.3389/froh.2022.859209 35464780 PMC9024052

[12] Ganesan SM Joshi V Fellows M Dabdoub SM Nagaraja HN O'Donnell B. A tale of two risks: smoking, diabetes and the subgingival microbiome. ISME J (2017) 11(9):2075–89. doi: 10.1038/ismej.2017.73 PMC5563960 28534880

[13] Demmer RT Breskin A Rosenbaum M Zuk A LeDuc C Leibel R. The subgingival microbiome, systemic inflammation and insulin resistance: The oral infections, glucose intolerance and insulin resistance study. J Clin Periodontology (2017) 44(3):255–65. doi: 10.1111/jcpe.12664 PMC5328907 27978598

[14] Shi B Lux R Klokkevold P Chang M Barnard E Haake S. The subgingival microbiome associated with periodontitis in type 2 diabetes mellitus. ISME J (2019) 14(2):519–30. doi: 10.1038/s41396-019-0544-3 PMC6976570 31673077

[15] Mesa F Magan-Fernandez A Castellino G Chianetta R Nibali L Rizzo M. Periodontitis and mechanisms of cardiometabolic risk: Novel insights and future perspectives. Biochim Biophys Acta Mol Basis Dis (2019) 1865(2):476–84. doi: 10.1016/j.bbadis.2018.12.001 30529255

[16] Rizzo M Cappello F Marfil R Nibali L Marino Gammazza A Rappa F. Heat-shock protein 60 kDa and atherogenic dyslipidemia in patients with untreated mild periodontitis: a pilot study. Cell Stress Chaperones (2012) 17(3):399–407. doi: 10.1007/s12192-011-0315-1 22215516 PMC3312963

[17] Nibali L Rizzo M Li Volti G D'Aiuto F Giglio RV Barbagallo I. Lipid subclasses profiles and oxidative stress in aggressive periodontitis before and after treatment. J Periodontal Res (2015) 50(6):890–6. doi: 10.1111/jre.12283 25994389

[18] Casarin RCV Barbagallo A Meulman T Santos VR Sallum EA Nociti FH. Subgingival biodiversity in subjects with uncontrolled type-2 diabetes and chronic periodontitis. J Periodontal Res (2013) 48(1):30–6. doi: 10.1111/j.1600-0765.2012.01498.x 22762355

[19] Takahashi N ST. Dipeptide utilization by the periodontal pathogens porphyromonas gingivalis, prevotella intermedia, prevotella nigrescens and fusobacterium nucleatum. Oral Microbiol Immunol (2002) 17(1):50–4. doi: 10.1046/j.0902-0055.2001.00089.x 11860556

[20] Barnes VM Ciancio SG Shibly O Xu T Devizio W Trivedi HM. Metabolomics reveals elevated macromolecular degradation in periodontal disease. J Dental Res (2011) 90(11):1293–7. doi: 10.1177/0022034511416240 21856966

[21] Giannobile WV Al-Shammari KF Sarment DP. Matrix molecules and growth factors as indicators of periodontal disease activity. Periodontol (2000) 31:125–34. doi: 10.1034/j.1600-0757.2003.03108.x 12656999

[22] Cueno ME Ochiai K. Gingival periodontal disease (PD) level-butyric acid affects the systemic blood and brain organ: Insights into the systemic inflammation of periodontal disease. Front Immunol (2018) 9. doi: 10.3389/fimmu.2018.01158 PMC5994410 29915575

[23] Eda H Zhang J Keith RH Michener M Beidler DR Monahan JB. Macrophage-colony stimulating factor and interleukin-34 induce chemokines in human whole blood. Cytokine (2010) 52(3):215–20. doi: 10.1016/j.cyto.2010.08.005 20829061

[24] Fine N Chadwick JW Sun C Parbhakar KK Khoury N Barbour A. Periodontal inflammation primes the systemic innate immune response. J Dental Res (2020) 100(3):318–25. doi: 10.1177/0022034520963710 33078669

[25] Hajishengallis G. Immunomicrobial pathogenesis of periodontitis: keystones, pathobionts, and host response. Trends Immunol (2014) 35(1):3–11. doi: 10.1016/j.it.2013.09.001 24269668 PMC3947349

[26] Kourtzelis I Li X Mitroulis I Grosser D Kajikawa T Wang B. DEL-1 promotes macrophage efferocytosis and clearance of inflammation. Nat Immunol (2018) 20(1):40–9. doi: 10.1038/s41590-018-0249-1 PMC6291356 30455459

[27] Zhu M Nikolajczyk BS. Immune cells link obesity-associated type 2 diabetes and periodontitis. J Dental Res (2014) 93(4):346–52. doi: 10.1177/0022034513518943 PMC3957341 24393706

[28] Blasco-Baque V Garidou L Pomié C Escoula Q Loubieres P Le Gall-David S. Periodontitis induced byPorphyromonas gingivalisdrives periodontal microbiota dysbiosis and insulin resistance *via* an impaired adaptive immune response. Gut (2017) 66(5):872–85. doi: 10.1136/gutjnl-2015-309897 PMC5531227 26838600

[29] Figueredo CM Lira-Junior R Love RM. T And b cells in periodontal disease: New functions in a complex scenario. Int J Mol Sci (2019) 20(16):3949. doi: 10.3390/ijms20163949 PMC6720661 31416146

[30] Lamont RJ Koo H Hajishengallis G. The oral microbiota: dynamic communities and host interactions. Nat Rev Microbiol (2018) 16(12):745–59. doi: 10.1038/s41579-018-0089-x PMC6278837 30301974

[31] Martinez-Herrera M Silvestre FJ Silvestre-Rangil J Bañuls C Rocha M Hernández-Mijares A. Involvement of insulin resistance in normoglycaemic obese patients with periodontitis: A cross-sectional study. J Clin Periodontol. (2017) 44(10):981–8. doi: 10.1111/jcpe.12773 28696512

[32] Maekawa T Krauss Jennifer L Abe T Jotwani R Triantafilou M Triantafilou K. Porphyromonas gingivalis manipulates complement and TLR signaling to uncouple bacterial clearance from inflammation and promote dysbiosis. Cell Host Microbe (2014) 15(6):768–78. doi: 10.1016/j.chom.2014.05.012 PMC4071223 24922578

[33] Geva-Zatorsky N Sefik E Kua L Pasman L Tan TG Ortiz-Lopez A. Mining the human gut microbiota for immunomodulatory organisms. Cell (2017) 168(5):928–43.e11. doi: 10.1016/j.cell.2017.01.022 28215708 PMC7774263

[34] Balmasova IP Lomakin YA Babaev EA Tsarev VN Gabibov AG Smirnov IV. “Shielding” of cytokine induction by the periodontal microbiome in patients with periodontitis associated with type 2 diabetes mellitus. Acta Naturae (2019) 11(4):79–87. doi: 10.32607/20758251-2019-11-4-79-87 31993238 PMC6977959

[35] Grauballe MB Østergaard JA Schou S Flyvbjerg A Holmstrup P. Effects of TNF-α blocking on experimental periodontitis and type 2 diabetes in obese diabetic zucker rats. J Clin Periodontology (2015) 42(9):807–16. doi: 10.1111/jcpe.12442 26257165

[36] Cutando A Montero J Gomez-de Diego R Ferrera MJ Lopez-Valverde A. Effect of topical application of melatonin on serum levels of c-reactive protein (CRP), interleukin-6 (IL-6) and tumor necrosis factor-alpha (TNF-a) in patients with type 1 or type 2 diabetes and periodontal disease. J Clin Exp Dentistry (2015) 7(5):e628–33. doi: 10.4317/jced.52604 PMC4663066 26644840

[37] Polak D Shapira L. An update on the evidence for pathogenic mechanisms that may link periodontitis and diabetes. J Clin Periodontology. (2018) 45(2):150–66. doi: 10.1111/jcpe.12803 29280184

[38] Jagannathan R Thayman M Rao SR. Proinflammatory (CD14+ CD16++) monocytes in type 2 diabetes mellitus patients with/without chronic periodontitis. J Dental Res J (2019) 16(2):95. PMC6364346 30820203

[39] Kumar PS Monteiro MF Dabdoub SM Miranda GL Casati MZ Ribeiro FV. Subgingival host-microbial interactions in hyperglycemic individuals. J Dental Res (2020) 99(6):650–7. doi: 10.1177/0022034520906842 32175785

[40] DeFuria J Belkina AC Jagannathan-Bogdan M Snyder-Cappione J Carr JD Nersesova YR. B cells promote inflammation in obesity and type 2 diabetes through regulation of T-cell function and an inflammatory cytokine profile. Proc Natl Acad Sci (2013) 110(13):5133–8. doi: 10.1073/pnas.1215840110 PMC3612635 23479618

[41] Muthukuru M Cutler CW. Resistance of MMP9 and TIMP1 to endotoxin tolerance. Pathog Dis (2014) 73(5):ftu003. doi: 10.1093/femspd/ftu003 25951835 PMC4849345

[42] Bascones-Martínez A Muñoz-Corcuera M Bascones-Ilundain J. Diabetes y periodontitis: una relación bidireccional. Medicina Clínica (2015) 145(1):31–5. doi: 10.1016/j.medcli.2014.07.019 25192582

[43] Sonnenschein SK Meyle J. Local inflammatory reactions in patients with diabetes and periodontitis. J Periodontology (2015) 69(1):221–54. doi: 10.1111/prd.12089 26252411

[44] Acharya AB Thakur S Muddapur MV Kulkarni RD. Cytokine ratios in chronic periodontitis and type 2 diabetes mellitus. Diabetes Metab Syndrome: Clin Res Rev (2017) 11(4):277–8. doi: 10.1016/j.dsx.2016.12.007 27989515

[45] Miranda TS Heluy SL Cruz DF da Silva HDP Feres M Figueiredo LC. The ratios of pro-inflammatory to anti-inflammatory cytokines in the serum of chronic periodontitis patients with and without type 2 diabetes and/or smoking habit. Clin Oral Investigations (2018) 23(2):641–50. doi: 10.1007/s00784-018-2471-5 29737428

[46] Chang E-J Lee SK Song YS Jang YJ Park HS Hong JP. IL-34 is associated with obesity, chronic inflammation, and insulin resistance. J Clin Endocrinol Metab (2014) 99(7):E1263–E71. doi: 10.1210/jc.2013-4409 24712570

[47] Long J Cai Q Steinwandel M Hargreaves MK Bordenstein SR Blot WJ. Association of oral microbiome with type 2 diabetes risk. J Periodontal Res (2017) 52(3):636–43. doi: 10.1111/jre.12432 PMC5403709 28177125

[48] Chee B Park B Bartold PM. Periodontitis and type II diabetes: a two-way relationship. Int J Evid Based Healthc. (2013) 11(4):317–29. doi: 10.1111/1744-1609.12038 24298927

[49] Wu Y-Y Xiao E Graves DT. Diabetes mellitus related bone metabolism and periodontal disease. Int J Oral Sci (2015) 7(2):63–72. doi: 10.1038/ijos.2015.2 25857702 PMC4817554

[50] Belkina AC Azer M Lee JJ Elgaali HH Pihl R Cleveland M. Single-cell analysis of the periodontal immune niche in type 2 diabetes. J Dental Res (2020) 99(7):855–62. doi: 10.1177/0022034520912188 PMC7313350 32186942

[51] Chen Q Ma X Li C Shen Y Zhu W Zhang Y. Enteric phageome alterations in patients with type 2 diabetes. Front Cell Infect Microbiol (2020) 10:575084. doi: 10.3389/fcimb.2020.575084 33552999 PMC7862107

[52] Yang K Niu J Zuo T Sun Y Xu Z Tang W. Alterations in the gut virome in obesity and type 2 diabetes mellitus. Gastroenterology (2021) 161(4):1257–69.e13. doi: 10.1053/j.gastro.2021.06.056 34175280

[53] Gastaldi G Goossens N Clément S Negro F. Current level of evidence on causal association between hepatitis c virus and type 2 diabetes: a review. J advanced Res (2017) 8(2):149–59. doi: 10.1016/j.jare.2016.11.003 PMC5272937 28149650

[54] Lin P-Y Chen S-C Lo T-C Kuo H-W. Dual infection with hepatitis b virus and hepatitis c virus correlated with type 2 diabetes mellitus. Exp Clin Endocrinol Diabetes (2020) 128(01):38–42. doi: 10.1055/a-0794-6135 30654388

[55] Karim S Mirza Z A Kamal M Abuzenadah A M Azhar E I Al-Qahtani M H. An association of virus infection with type 2 diabetes and alzheimer’s disease. Neurological Disorders-Drug Targets (2014) 13(3):429–39. doi: 10.2174/18715273113126660164 24059298

[56] Xu Z Shi L Wang Y Zhang J Huang L Zhang C. Pathological findings of COVID-19 associated with acute respiratory distress syndrome. Lancet Respir Med (2020) 8(4):420–2. doi: 10.1016/S2213-2600(20)30076-X PMC7164771 32085846

[57] de Almeida Pinheiro T Barcala-Jorge AS Andrade JMO de Almeida Pinheiro T Ferreira ECN Crespo TS. Obesity and malnutrition similarly alter the renin–angiotensin system and inflammation in mice and human adipose. J Nutr Biochem (2017) 48:74–82. doi: 10.1016/j.jnutbio.2017.06.008 28779634

[58] Kaparianos A Argyropoulou E. Local renin-angiotensin II systems, angiotensin-converting enzyme and its homologue ACE2: their potential role in the pathogenesis of chronic obstructive pulmonary diseases, pulmonary hypertension and acute respiratory distress syndrome. Curr medicinal Chem (2011) 18(23):3506–15. doi: 10.2174/092986711796642562 21756232

[59] Hotamisligil GS. Inflammation and metabolic disorders. Nature (2006) 444(7121):860–7. doi: 10.1038/nature05485 17167474

[60] Drucker DJ. Coronavirus infections and type 2 diabetes–shared pathways with therapeutic implications. Endocrine Rev (2020) 41(3):bnaa011. doi: 10.1210/endrev/bnaa011 32294179 PMC7184382

[61] Contreras A Botero JE Slots J. Biology and pathogenesis of cytomegalovirus in periodontal disease. Periodontol 2000. (2014) 64(1):40–56. doi: 10.1111/j.1600-0757.2012.00448.x 24320955 PMC7167941

[62] Imai K Yamada K Tamura M Ochiai K Okamoto T. Reactivation of latent HIV-1 by a wide variety of butyric acid-producing bacteria. Cell Mol Life Sci (2012) 69(15):2583–92. doi: 10.1007/s00018-012-0936-2 PMC11114855 22322557

[63] Daigle D Gradoville L Tuck D Schulz V Wang'ondu R Ye J. Valproic acid antagonizes the capacity of other histone deacetylase inhibitors to activate the Epstein-barr virus lytic cycle. J Virol (2011) 85(11):5628–43. doi: 10.1128/JVI.02659-10 PMC3094991 21411522

[64] Casarin RCV Duarte PM Santos VR Lima JA Gagnon G Casati MZ. Influence of glycemic control on Epstein-bar and cytomegalovirus infection in periodontal pocket of type 2 diabetic subjects. Arch Oral Biol (2010) 55(11):902–6. doi: 10.1016/j.archoralbio.2010.07.009 20728869

[65] Abooj J Varma SA. Prevalence of herpes virus in chronic periodontitis patients with and without type 2 diabetes mellitus: A clinico-microbiological study. J Oral Maxillofac Pathology: JOMFP (2021) 25(1):141. doi: 10.4103/jomfp.JOMFP_154_20 PMC8272489 34349425

[66] Roganović JR. microRNA-146a and-155, upregulated by periodontitis and type 2 diabetes in oral fluids, are predicted to regulate SARS-CoV-2 oral receptor genes. J periodontology (2021) 92(7):e35–43. doi: 10.1002/JPER.20-0623 33336412

